# An exploratory analysis of bezisterim treatment associated with decreased biological age acceleration, and improved clinical measure and biomarker changes in mild-to-moderate probable Alzheimer's disease

**DOI:** 10.3389/fnins.2025.1516746

**Published:** 2025-05-02

**Authors:** Christopher L. Reading, Jiayan Yan, Marcia A. Testa, Donald C. Simonson, Hira Javaid, Lisa Schmunk, Daniel E. Martin-Herranz, Robert Brooke, Juozas Gordevicius, Jeffrey Zhang, Harvey Yuan, Clarence Ahlem, Lixia Wang, Penelope Markham, Nily Osman, Stephen O'Quinn, Joseph Palumbo

**Affiliations:** 1 BioVie Inc., Carson City, NV, United States; 2 Department of Biostatistics, Harvard T. H. Chan School of Public Health, Boston, MA, United States; 3 Division of Endocrinology, Diabetes, and Hypertension, Brigham and Women's Hospital, Harvard Medical School, Boston, MA, United States; 4 Hurdle.bio/Chronomics Ltd., London, United Kingdom; 5 Epigenetic Clock Development Foundation, Torrance, CA, United States; 6 Princeton Pharmatech, Princeton, NJ, United States

**Keywords:** Alzheimer's, neuroinflammation, insulin, aging, DNA methylation, bezisterim, cognition, dementia

## Abstract

**Introduction:**

Aging is the primary risk factor for sporadic Alzheimer's disease. Chronic low-grade inflammation associated with aging drives cognitive impairment through multiple mechanisms involving oxidative stress, insulin resistance, and dysregulation of metabolic, immunologic, and hematologic systems.

**Methods:**

In a 7-month, randomized, double-blind, placebo-controlled trial (NCT04669028), we investigated the safety and activity of bezisterim, a first-in-class, oral, blood–brain barrier–permeable, anti-inflammatory agent on cognitive, molecular, biochemical, physiological, and biological aging parameters in a subset of 50 mild-to-moderate probable Alzheimer's disease participants. These participants had source-document-verified clinical measures and samples, and they completed the protocol. This study focuses on epigenetic, metabolic, biomarker, and cognitive measures in the exploratory biomarker population that completed the protocol.

**Results:**

Bezisterim was associated with non-significant directional improvements in multiple measures of cognitive and functional performance compared to placebo, with correlations to biological age (determined by DNA methylation “clocks”) and to metabolism, inflammation, and dementia biomarkers. In addition, clinical measures correlated with the extent of DNA methylation of certain cytosine-phosphate-guanine (CpG) sites in genes associated with metabolic inflammation and neurodegeneration.

**Discussion:**

The results suggest the possible use of bezisterim to target the multifactorial processes underlying dementia.

**Clinical trial registration:**

https://clinicaltrials.gov/study/NCT04669028, Identifier: NCT04669028.

## Introduction

Converging evidence indicates that cognitive decline in Alzheimer's disease (AD) is driven by a persistent and complex pathophysiological process, including metabolic dysregulation (Johnson et al., 2020). Beyond the well-characterized neurofibrillary tangles, dystrophic neurites, and amyloid-beta (Aβ) deposits, AD-associated pathology includes neuroinflammation, neurodegeneration, apoptosis, DNA damage, mitochondrial malfunction, compromised energy metabolism, and chronic oxidative stress. Indeed, metabolic dysfunction is considered a leading cause and a hallmark of AD that is also apparent decades prior to disease manifestation (Knopman et al., 2021; Marder et al., 2014).

Neuroinflammation and insulin resistance contribute to neurological dysfunction and neurodegeneration (Reading et al., 2021). Prion-associated misfolding of Aβ1-42 and phospho-Tau (pTau) proteins leads to aggregates that are recognized by molecular pattern recognition receptors (Chiarini et al., 2020; Salminen et al., 2009). This leads to the activation of nuclear factor-kappa B (NF-κB), proinflammatory chemokines, and cytokines, resulting in astrogliosis, proinflammatory microglial transition, and neuroinflammation (Okun et al., 2009; Fiebich et al., 2018). These stepwise inflammatory responses increase IκB kinase β (IKKβ) and c-Jun N-terminal kinase (JNK) serine phosphorylation of insulin receptor substrate 1/2 (IRS1/2), decreasing insulin and insulin-like growth factor 1 (IGF1) signaling and glucose uptake, leading to neuronal dysfunction and death (Reading et al., 2021). In AD, where microglial biology is likely a driver of pathogenesis, inflammatory stimuli, such as fibrillar amyloid, activate toll-like receptor 4 (TLR4), which then triggers the extracellular signal-regulated kinases 1/2 (ERK1/2)—the proteins that are central to microglial dysfunction (Chen et al., 2021).

Bezisterim (17alpha-ethynyl-androst-5-ene-3beta, 7beta, 17beta-triol [formally HE3286/NE3107]) (Ahlem et al., 2011b) is an analog of 5-androstene-3beta,7beta,17beta-triol (βAET), an adrenal sterol metabolite found in young adult humans at picomolar concentrations that decreases with age (Ahlem et al., 2011a; Auci et al., 2011). βAET is a neurosteroid produced in the hippocampus by the dentate gyrus-defining Cyp7b 17-hydroxylation of sterol intermediates (Rose et al., 2001; Thompson et al., 2008; Yau et al., 2003; Maehata et al., 2020). Importantly, βAET inhibits inflammation-activated NF-κB–mediated transcription (Stickney et al., 2011) (and unpublished observations). Conversely, low-grade chronic inflammation can result in 17-hydroxyl oxidation, forming inactive 7-hydroxy-dehydroepiandrosterone (DHEA) (Stickney et al., 2011). C-17 ethynylation of βAET to form bezisterim preserves the 17β-hydroxyl structure that can inhibit inflammatory NF-κB transcription, and confers oral bioavailability and blood-brain barrier permeability (Ahlem et al., 2011b). Bezisterim binds ERK1/2, as well as the ERK-binding partners low-density lipoprotein receptor-related protein 1 (LRP1), ribosomal S6 kinase 2 (RSK2), and sirtuin 2 (SIRT2) (Reading et al., 2012). Bezisterim inhibits TLR4- and tumor necrosis factor (TNF)-stimulated NF-κB activation and signal transduction by decreasing ERK phosphorylation (Wang et al., 2010; Lu et al., 2010) and it may selectively interact with ERK in the NF-κB1/MAP3K8/MEK/ERK scaffold required for TNF transcription (MAP3K8 refers to mitogen-activated protein kinase kinase kinase 8; MEK refers to mitogen-activated protein kinase kinase) (Dumitru et al., 2000), which leads to decreased expression of proinflammatory cytokines and chemokines (Lu et al., 2010). Bezisterim inhibited inflammatory TNF activation and improved insulin sensitivity in rodent models (Wang et al., 2010; Lu et al., 2010), and in human participants with obesity-induced glucose intolerance or type-2 diabetes (Reading et al., 2013a,b). Bezisterim showed improvements in animal neurodegeneration models (Khan et al., 2014; Lambert et al., 2017), including pro-motor effects and decreased neurodegeneration in rodent and primate Parkinson's disease (PD) models (Nicoletti et al., 2012; Philllipens et al., 2023), as well as pro-motor activity in a phase 2a study (NCT05083260) in patients with PD, both with and without levodopa (Aldred et al., 2023a,b). In a 14-week, open-label, phase 2a study (NCT05227820) of mild cognitive impairment and mild AD, bezisterim improved neurological assessments, neuroimaging, and precuneus glutathione levels while decreasing cerebrospinal fluid (CSF) pTau (Haroon et al., 2024).

Inflammation may also alter DNA methylation patterns associated with diseases of aging (Schmunk et al., 2023). Furthermore, biological age, as calculated using epigenetic aging clocks, is associated with changes in metabolic and inflammatory markers (Schmunk et al., 2023; Hannum et al., 2013; Horvath et al., 2018; Levine et al., 2018; Lu et al., 2019). We now report analyses from an exploratory biomarker sub-population in a double-blind, randomized, placebo-controlled study, NM101 (NCT04669028), in patients with mild-to-moderate AD. Details of the NM101 protocol were previously published (Reading et al., 2021).

## Methods

### Study design and participants

In this double-blind, randomized, placebo-controlled, parallel group, multicenter phase-3 trial, we evaluated the safety, tolerability, and efficacy of bezisterim in participants with mild-to-moderate probable AD. Institutional review board approval was obtained from ADVARRA (MOD01862543). Participants were informed of the treatment and the samples collected during the trial in a written informed consent document. The study enrolled participants at 39 sites across the United States from August 2021 through October 2022, and randomly assigned participants 1:1 to receive 20-mg BID bezisterim or placebo during the 30-week treatment period. All study personnel, sponsors, and participants were blinded to drug or placebo assignment prior to database lock. The study consisted of a screening period of up to 6 weeks, a treatment period of 30 weeks, and a 4-week follow-up period.

Eligible participants were aged 60 to 85 years and had mild-to-moderate probable AD, defined by a Clinical Dementia Rating (CDR) Standard Global Score of 1 to 2, inclusive; had a Mini-Mental State Examination (MMSE) score of ≥14 and ≤ 24 at both screening and baseline visits (difference in scores between screening and baseline < 3 points); had a historical magnetic resonance imaging (MRI) or computed tomography (CT) scan of the brain on file no earlier than AD diagnosis that did not exhibit features of another potential pathobiology that could better account for the cognitive disorder; and met the National Institute on Aging and Alzheimer's Association (NIA-AA, 2011) criteria of all-cause dementia and probable AD. The participants were required to have a modified Hachinski Ischemic Scale score of ≤ 4, and they were required to identify a primary caregiver or study partner prior to enrollment in the study to assist with study participation.

### Primary and secondary endpoints

The primary endpoints of this study were the change from baseline (V2) to week 30 (Visit 10/early termination) in the Alzheimer's Disease Assessment Scale-Cognition 12 (ADAS-Cog12) and the Alzheimer's Disease Cooperative Study-Clinical Global Impression of Change (ADCS-CGIC) scores. Secondary endpoints included: (1) neuropsychiatric and behavioral deficits as measured by changes in MMSE, Alzheimer's Disease Composite Score (ADCOMS), Clinical Dementia Rating Sum of Boxes (CDR-SB), and Neuropsychiatric Index (NPI) scores; (2) functional outcome as determined by change in Alzheimer's Disease Cooperative Study-Activities of Daily Living (ADCS-ADL); and (3) glycemic control as measured by changes in fasting insulin levels using Homeostatic Model Assessment-Insulin Resistance (HOMA2-IR), Mean Amplitude of Glycemic Excursion (MAGE), postprandial glucose, fructosamine level, and fasting blood glucose level.

After this study was underway, we discovered from a separate study that bezisterim may have important effects on aging clocks (Reading et al., 2023). As a result, we added exploratory endpoints to assess bezisterim's effects on epigenetic aging clocks using DNA methylation analysis, resource use as measured by the Resource Utilization in Dementia—Lite (RUD Lite); the influence of apolipoprotein E (ApoE) genotype classified as ApoE positive vs negative on the primary and selected secondary outcomes; the exploration of the effects on plasma levels of inflammatory- and/or neurodegeneration-related biomarkers; neuroimaging scan results of volumetric magnetic resonance imaging (vMRI) and fluorodeoxyglucose positron emission tomography (FDG-PET); and APTUS test and epigenetic analysis using DNA methylation.

Safety endpoints included the incidence and severity of treatment-emergent adverse events (TEAEs), vital signs, physical examinations, 12-lead electrocardiogram assessments, clinical laboratory assessments (hematology, chemistry, and urinalysis), and Columbia-Suicide Severity Rating Scale (C-SSRS) scores.

**Figure 1:**
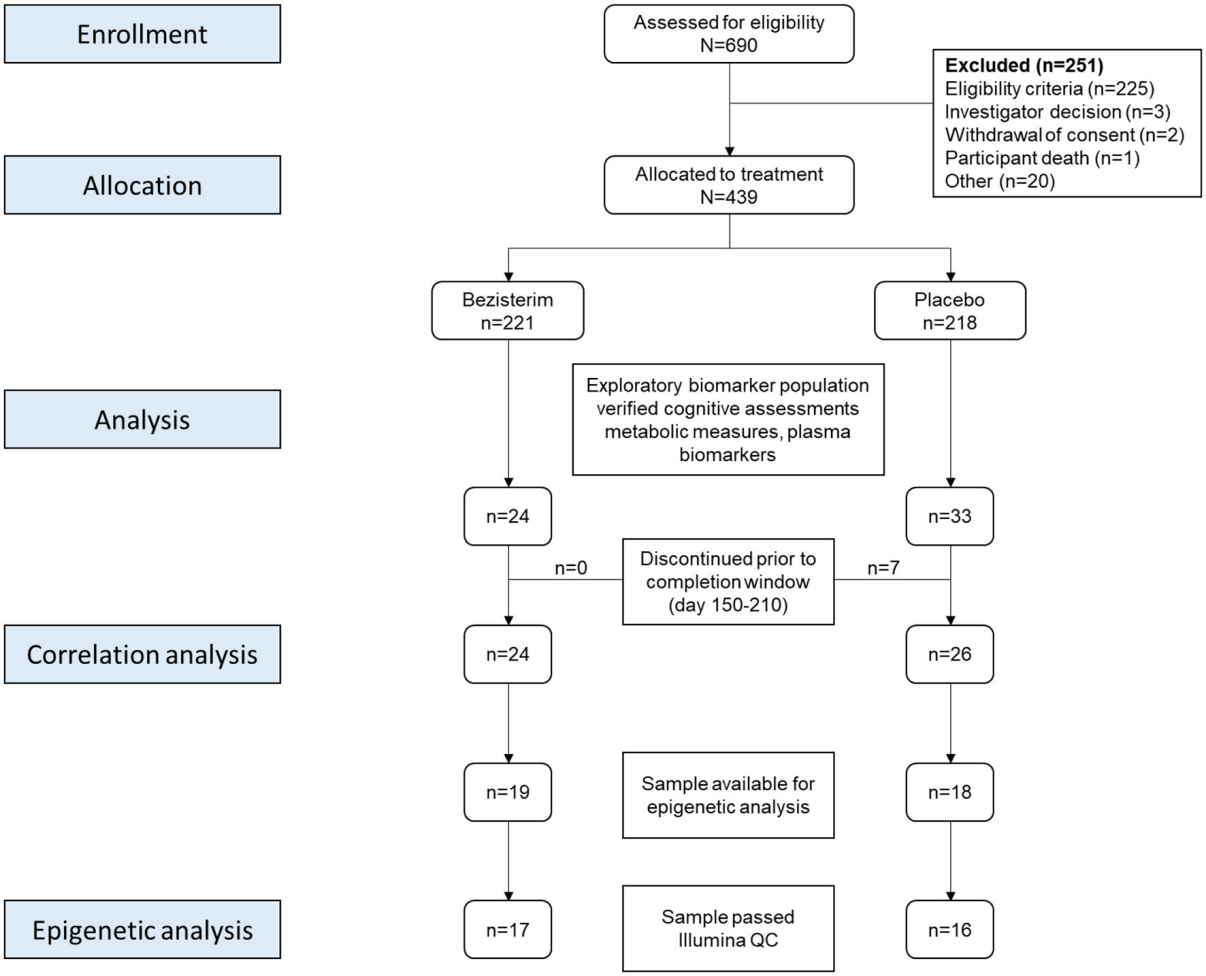
CONSORT flow diagram for the patients included in correlation analyses and DNA methylation analyses from the NM101 trial. A total of 690 were screened for eligibility from August 2021 through October 2022 at US clinical sites. After exclusions, 439 Participants were randomly assigned (1:1) to receive 20-mg bezisterim or placebo during the 30-week treatment period. All study personnel, sponsors, and participants were blinded to drug or placebo assignment prior to database lock. In March 2023, the protocol was amended to include samples for analysis of plasma pTau217, GFAP, NfL, Aβ40, Aβ42, adiponectin, leptin, and blood DNA methylation, allowing for exploratory biomarker analyses and their correlations with neurocognitive assessments. A total of 57 participants underwent source document-verified cognitive assessments, metabolic measures, inflammatory measures, and plasma biomarkers. Seven placebo participants discontinued prior to the completion window (day 150–210), leaving 50 for correlation analyses. Samples for DNA methylation were available for 37 of these participants, of whom 33 passed Illumina QC. CONSORT, Consolidated Standards of Reporting Trials; pTau217, phospho-217 Tau protein; GFAP, glial fibrillary acidic protein; NfL, neurofilament light chain; Aβ40, amyloid beta 40; Aβ42, amyloid beta 42.

Consolidated Standards of Reporting Trials (CONSORT) information for the exploratory biomarker population is presented in Figure 1.

Additional CONSORT information for the overall trial is presented in Supplementary File S1. There were important protocol amendments with changes to safety, eligibility, or study endpoints. Version 1.1 (14 April 2021) excluded females of child-bearing potential, as bezisterim did not have supporting reproductive toxicology data. Version 1.2 (20 April 2022) added the exclusion of recent coronavirus disease 2019 (COVID-19) infection that could impact neurological testing. Version 1.2 also added the inclusion criterion of historical MRI or CT scan to confirm absence of pathobiology that might account for the cognitive disorder and historical evidence of cognitive impairment or dementia diagnosis, and removal of inclusion requirement for evidence of Aβ, since bezisterim was not an anti-amyloid treatment, and enrollment was very challenging for Aβ-positive population. Version 1.3 (05 December 2022) revised continuous glucose monitoring to be optional, as it was perceived that it might be limiting enrollment. Version 1.4 (10 March 2023) added pTau, glial fibrillary acidic protein (GFAP), neurofilament light chain (NfL), adiponectin, leptin, and DNA methylation analysis to biomarkers to better characterize response correlations. Version 2.0 (12 May 2023) changed the primary endpoint to co-primary endpoints of ADAS-Cog12 and ADCS-CGIC to a single primary endpoint of CDR-SB, based on recent endpoints used in AD trials. Version 3.0 (09 October 2023) changed the primary efficacy co-primary endpoints of ADAS-Cog12 and ADCS-CGIC, based on discussions with the US Food and Drug Administration (FDA) that a single primary endpoint would not be adequate for accelerated approval for agents without an established biomarker. The protocol was also modified to include the statistical considerations in the statistical analysis plan prior to unblinding, including definitions of the modified intent-to-treat (mITT) and per-protocol populations, as well as clarifications of the secondary, tertiary, and exploratory endpoints. Version 3.1 (23 October 2023) was modified to change the co-primaries to CDR-SB and ADAS-Cog12 to be more in line with recent approvals based on CDR-SB.

In version 3.1, the sample size was increased to approximately 400 participants to be randomized into the study with a 1:1 treatment ratio to have at least 80% power to detect the treatment difference for the co-primary endpoints. This was estimated from the CDR-SB change from baseline results in the mild AD patients of our open-label study (MMSE >20, *n* = 18). Conservatively, we assume that the treatment group would observe a −0.46 CDR-SB improvement (two-thirds of a −0.7 CDR-SB improvement observed in the open-label study). Assuming a placebo effect contributed approximately −0.23 of the −0.46 CDR-SB point improvement and that the placebo would decline (CDR-SB increase) by 0.15 during the study, the net assumed treatment difference was 0.38. Assuming the same standard deviation (SD) observed in the open-label study, SD = 1.1. As for ADAS-Cog12, the samples were estimated to have at least 80% power to detect a 2.1-point difference between bezisterim and placebo, assuming a SD of 7. The placebo is expected to have a similar SD.

All changes to the study were reviewed by the Sponsor and the contract research organization, who were blinded to treatment allocations. Although an interim analysis was originally planned, the rapid enrollment following the protocol modifications made this impractical, and the interim analysis was eliminated.

The exploratory biomarker population consisted of participants who had source document-verified clinical measures, completed the study without serious protocol violations, and had samples collected that were defined in the protocol amendment in March 2023, allowing for analysis of blood AD-associated biomarkers and DNA methylation. The exploratory biomarker population was not sufficient to meet protocol-defined endpoints, including neurocognitive assessments, clinical, metabolic, inflammatory, epigenetic, and dementia measures. This did not preclude exploratory analyses conducted in the biomarker population as described in the “Statistical analysis” section.

### Sample collection and processing

For correlations of Horvath Skin Blood Clock (SBC) with neurological assessments, DNA samples were collected at study completion in ethylenediaminetetraacetic acid tubes and stored frozen. The Illumina Array processing and analyses were performed by the Epigenetic Clock Development Foundation (ECDF) for DNA methylation analyses. Blinded participants' DNA samples were randomized into arrays for DNA methylation analysis in 2 batches of completed participants.

Data for differences between biological age and chronologic age, as well as for age acceleration differences, were analyzed using a single DNA methylation array from DNA isolated from a single blood draw at the completion visit for each subject. Hematology analyses for each individual are from a single blood draw at completion. DNA methylation analysis for cell “clocks” are from the same single tube and DNA sample used for the biological age analyses. Laboratory values are from single samples from single blood draws at completion, but from different tubes depending on the test performed. For pTau217, NfL, GFAP, Aβ40, and Aβ42, data were from duplicate samples from the same tube for each subject, measured at the same time.

### Statistical analysis

For Pearson correlation analysis, exploratory correlation coefficient r and *p*-values were computed to determine the significance of the correlation at a 90% confidence interval. Exploratory *p*-values were not corrected for multiple comparisons. Neurological assessments, as well as biological aging, metabolic, inflammatory, and dementia biomarkers, were assembled into csv files along with data from the ECDF (https://clockfoundation.org/), biostatistics NM101 database, LabConnect NM101 laboratory database, and Quanterix, Inc. Data for biomarkers Aβ40, Aβ42, pTau217, GFAP, and NfL were assessed for the 50 participants in the exploratory biomarker population who completed the study. These data were used for correlation matrices. Missing values were imputed using the BH Bayesian R code (www.R-project.org). Outliers for the clinical measures were detected using the Grubbs test at, α = 0.05. If an influential observation was detected for a clinical measure, it was removed, and the correlation matrix was imputed to avoid the false outlier correlation. The correlation matrix was normalized using the Z-score R code. Clinical Pearson correlations and *p*-values were identified using the Prism correlation matrix. For exploratory analyses, the alpha level was set at *p* < 0.05. EPIC v2.0 analysis data were preprocessed and converted to beta values using the MinFi R code and normalized using Z′-values. Values for the biological aging clocks that passed ECDF quality control (QC) were assembled into a data matrix, which included results from the 33 completion samples. Correlations of the clinical measure matrix for participants with SBC DNA methylation data were analyzed using Prism. Distributions and Welch *t*-tests of differences for SBC deltaAge (dAge, the difference between biological age and chronological age), PhenoAge, GrimAge, and AgeHannum clocks were analyzed using the ECDF-reported values normalized to Z-scores. Data for the InflammAge clock were derived by Hurdle, as were corrections for control probes and age acceleration for all five biological age clocks. Analyses of correlations between hematology clock (lymphocyte, granulocyte, and monocyte predictions) and hematology laboratory values were performed in Prism, and the *p*-values were not corrected for multiple comparisons. Significant correlations of clinical measures with DNA methylation sites were corrected for multiple comparisons using False Discovery Rate < 0.05 to determine adjusted *p*-values < 0.05 using R code. Individual cytosine-phosphate-guanine (CpG) beta value Z′-score correlations with individual clinical measures were performed in R code using Pearson and false discovery rate (FDR) programs to derive r and adjusted *p*-values.

Treatment groups were compared with respect to participant demographics and baseline characteristics, and differences in continuous variables were tested using Welch *t*-test, and differences in categorical variables differences were tested using the chi-square test.

For the per-protocol population, Bayesian Pearson correlation analysis was performed to analyze the association between neurological assessments and metabolic, inflammatory, and dementia biomarkers. Correlation analyses are from single laboratory or clinical measurements at the completion visit. Laboratory values are from single samples obtained from a single blood draw at completion, but collected from different tubes depending on the test performed. pTau217, NfL, GFAP, Aβ40, and Aβ42 data were from duplicate samples that were taken from the same tube for each subject.

DNA methylation data were generated using the Illumina Infinium Methylation EPIC v2.0 array (Illumina, Inc.). Epigenetic clock results were obtained for SBCAge (Horvath et al., 2018), PhenoAge (Levine et al., 2018), GrimAge (Lu et al., 2019), and Hannum clocks (Hannum et al., 2013), and cell composition proxies from ECDF, and for the InflammAge clock (Schmunk et al., 2023) by Hurdle, using published methods.

The difference between biological age and chronological age (dAge) was calculated across all epigenetic clocks. dAge comparisons were performed using Welch 2-sample *t*-tests for both bezisterim- and placebo-group participants. Age acceleration was calculated as the residuals from the following linear model: Biological_Age ~ Chronological_Age.

The analysis verified that technical variation and/or batch effects were not influencing the age acceleration result. For this, principal component analysis was performed on the measurements from the array control probes as previously described (Martin-Herranz et al., 2019), and the first two principal components (PC1/PC2) were added to the following linear model as covariates before calculating the residuals: Biological_Age ~ Chronological_Age + PC1 + PC2.

Principal component analyses were performed in Prism, using correlation analyses from single laboratory or clinical measurements at the completion visit.

For the per-protocol population, dAge (as determined by the Horvath SBC and chronological age) was analyzed for bezisterim and placebo group participants. Bayesian Pearson correlation analysis and principal component analysis were performed to analyze the association between dAge and neurological assessments.

DNA methylation at the individual CpG level was assessed for correlations with clinical measures, with a false discovery rate < 0.05 using Bayesian Pearson correlation.

## Results

### Participants

The key eligibility criteria for participants are described in the “Methods” section and listed in Table 1. The disposition of the exploratory biomarker population participants is summarized in Figure 1. A total of 690 participants were screened for eligibility from August 2021 through October 2022 at the US clinical sites. After exclusions, 439 Participants were randomly assigned (1:1) to receive 20-mg bezisterim or placebo during the 30-week treatment period. All study personnel, sponsors, and participants were blinded to drug or placebo assignment prior to database lock. In March 2023, the protocol was amended to include samples for analysis of plasma pTau217, GFAP, NfL, Aβ40, Aβ42, adiponectin, leptin, and blood DNA methylation (based on phase 2a data), to allow for exploratory biomarker analyses and their correlations with neurocognitive assessments. A total of 57 participants underwent source document-verified cognitive assessments, metabolic measures, and inflammatory measures, as well as plasma biomarkers analysis. Seven placebo participants discontinued prior to the completion window (days 150–210), leaving 50 for correlation analyses.

**Table 1:** Key eligibility characteristics.

**Inclusion**	**Criteria**
Sex	Male or female
Age	60–85 years
Probable AD	NIA-AA 2011 criteria of all cause dementia and probable AD
CDR global score	1–2
MMSE	14–24 inclusive
MRI or CT	No evidence of neuropathology inconsistent with a diagnosis of AD
Caregiver/Study partner	Familiar with their status and would be able to support the participant for the duration of the study
**Exclusion**	**Criteria**
Imaging	Prior brain imaging inconsistent with probable AD
Stroke	History of a stroke that resulted in a cognitive or motor deficit, MRI, or CT evidence of a moderate or large cerebral infarct
Laboratory tests	Clinically relevant abnormalities include vitamin B12, thyroid function, severe anemia, and electrolyte abnormalities
Insulin treatment	Type-1 diabetes or type-2 diabetes requiring insulin treatment or the need to use continuous glucose monitoring
Seizure	Epilepsy or seizure disorder requiring ongoing treatment, or any seizure or loss of consciousness within 12 months prior to screening

NIA-AA, National Institute on Aging–Alzheimer's Association.

Demographics and baseline characteristics of the per-protocol population are presented in Table 2. These parameters were well balanced overall. Per-protocol participants randomized to bezisterim were confirmed to have the drug in their plasma at the completion of their visit.

**Table 2:** Demographics and baseline characteristics (per-protocol completers).

**Characteristic**	**Placebo**	**Bezisterim**	***p*-value^a^**
	**(*n* = 26)**	**(*n* = 24)**	
Gender, *n* (%)			0.59
Male	12 (46)	15 (63)	
Female	14 (54)	9 (38)	
Age, years, mean (range)	76 (70–80)	77 (63–81)	0.73
Race, *n* (%)			0.67
Caucasian	22 (85)	22 (92)	
Non-Caucasian	4 (15)	2 (8.3)	
Black	2 (7.7)	2 (8.3)	
Asian	1 (3.8)	0	
Not Identified	1 (3.8)	0	
APOE, *n* (%)			0.17
APOE4–	15 (58)	9 (38)	
APOE4+	11 (42)	15 (63)	
**Neurological assessments**			
ADCOMS composite Missing	0.80 (0.64–1.0) 2	0.80 (0.64–1.5) 0	0.65
ADL total score	61 (46–68)	64 (47–69)	0.70
CDR global score, *n* (%)			0.73
1.0	20 (77)	20 (83)	
2.0	6 (23)	4 (17)	
CDR-SB total score	6 (5.4–7.9)	6 (6.0–7.8)	0.49
CGIC	NA (defined 4.0)	NA (defined 4.0)	
ADAS2 Cog12 total Missing	34 (24–40) 1	33 (23–36) 0	0.75
GST total score Missing	0.31 (−0.48–1.7) 1	0.17 (−0.64–1.1) 0	0.57
MMSE total score	21 (17–24)	20 (19–24)	0.54
Mild: moderate			>0.999
>20, *n* (%)	15 (58)	14 (58)	
≤ 20, *n* (%)	11 (42)	10 (42)	
NPI total score frequency × severity Missing	4 (1–9) 3	5 (0–9) 1	0.78
**Metabolic parameters**			
Cholesterol, mg/dL	173 (149–200)	184 (165–212)	0.26
Fructosamine, μmol/L	240 (229–249)	240 (224–264)	0.67
Glucose, mg/dL	94 (85–102)	96 (89–109)	0.33
HOMA2-IR	1.0 (0.68–1.5)	0.86 (0.60–1.6)	0.82
HOMA2-%B	86 (70–105)	72 (61–115)	0.35
HOMA2-%S	100 (65–148)	117 (62–167)	0.82
Insulin, μU/mL	7.4 (5.2–11.5)	6.6 (4.6–12.9)	0.81
Systolic BP, mm Hg	132 (121–146)	135 (123–177)	0.80
Diastolic BP, mm Hg	76.5 (69–81)	81.5 (73–86)	0.18
Triglycerides, mg/dL	100 (75–126)	101 (77–152)	0.67
Weight, lbs	169 (143–193)	162 (140–178)	0.31
WHR Missing	0.95 (0.88–1.0) 1	0.2 (0.83–1.1) 2	0.27
**Inflammatory biomarkers**			
C1q, mg/dL Missing	20 (18–23) 1	21 (20–24) 0	0.29
CRP, mg/L Missing	1.3 (0.50–2.5) 3	1.2 (0.48–2.2) 2	0.84
MCP1, pg/mL Missing	454 (367–556) 1	453 (288–646) 0	0.78
% Monocytes (of WBC)	8 (6.0–9.0)	7 (6.3–8.8)	0.82
TNF, pg/mL Missing	1.4 (1.0–1.8) 7	1.5 (1.3–1.7) 4	0.47
RANTES, ng/mL Missing	17 (14–19) 1	19 (13–31) 0	0.17
**Dementia biomarkers**			
Aβ42/40 ratio Missing	0.06 (0.05–0.06) 2	0.06 (0.05–0.07) 2	0.61
GFAP, pg/mL Missing	303 (196–388) 2	306 (248–393) 2	0.48
NfL, pg/mL Missing	37 (28–57) 3	38 (26–53) 1	0.83
pTau217, pg/mL Missing	1.2 (0.56–1.8) 4	1.2 (0.87–1.6) 1	0.52

Values are median (IQR) unless specified as n (%) or mean (range).

^a^p-Values were derived from non-parametric Mann–Whitney test for continuous variables and chi-squared test or Fisher's Exact test for discrete variables.

%B, percent beta cell function; %S, percent insulin sensitivity; Aβ42/40, amyloid beta peptide 1–42/1–40 ratio; ADCOMS, Alzheimer's Disease Composite Score; ADL, Alzheimer's Disease Cooperative Study Activities of Daily Living; BP, blood pressure; CDR, Clinical Dementia Rating; CDR-SB, Clinical Dementia Rating Sum of Boxes; CGIC, Alzheimer's Disease Cooperative Study Clinicians Global Impression of Change; ADAS-Cog12, Alzheimer's Disease Assessment Scale-Cognition 12; CRP, C-reactive protein; C1q, complement component 1q; GFAP, glial fibrillary acidic protein; Glucose, fasting plasma glucose; GST, Global Statistical Test of Cog12 and CDR-SB; HOMA2-IR, Homeostatic Model Assessment 2 Insulin Resistance; Insulin, fasting plasma insulin; MCP1, monocyte chemoattractant protein 1; MMSE, Mini-Mental State Exam; NfL, neurofilament light chain; NPI, Neuropsychiatric Index 10 Frequency x Severity Total Score; pTau217, tau protein phosphorylated on serine 217; RANTES, regulated on activation, normal T cell expressed and secreted; SB, sum of boxes; SEM, standard error of the mean; TNF, tumor necrosis factor; Weight (kg); WHR, waist/hip ratio.

After all participants were enrolled and following baseline visits, the protocol was amended to collect samples at study completion (days 150–210) for DNA methylation analyses based on the phase 2a epigenetics observations. Completion visit DNA samples were available for 37 participants; however, 4 samples failed QC for the epigenetics testing data, leaving 33 participants (17 from the bezisterim and 16 from the placebo groups) for DNA methylation analyses.

### Exploratory efficacy endpoints (per-protocol population)

#### Primary endpoint

**Figure 2:**
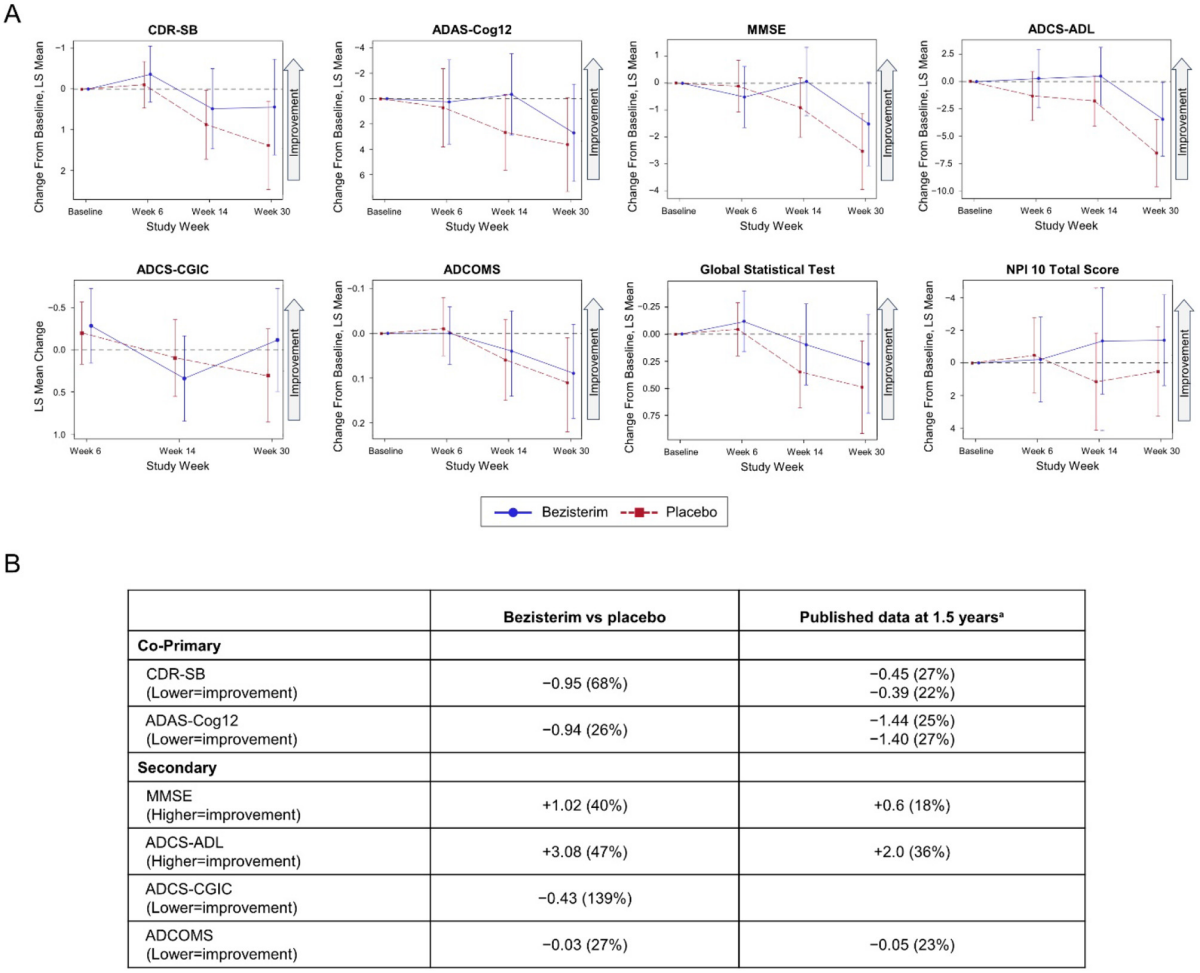
Primary and secondary endpoints for per-protocol population. **(A)** Scores for bezisterim and placebo from baseline through week 30. **(B)** Mean differences for bezisterim vs. placebo at week 30 in the context of results reported in clinical trials of approved AD medications. ^a^These data are from (van Dyck et al. 2023) and Budd Haeberlein et al. (2022).

The pattern of response in the 2 co-primary endpoints for the 50 subject per-protocol population suggested non-significant improvements associated with bezisterim on CDR-SB, ADAS-Cog12, and the Global Statistical Test (GST) of ADAS-Cog12 at all timepoints from baseline through the week 30 endpoint, with least squares mean (LS mean) differences of −0.95 (95% CI: −2.51, 0.61) from placebo for CDR-SB; −0.94 (95% CI: −6.16, 4.28) for ADAS-Cog12; and −0.22 (95% CI: −0.83, 0.39) for GST at week 30 (Figure 2).

#### Key secondary endpoints

There were 5 key secondary efficacy endpoints: ADCOMS, ADL, CGIC, MMSE, and NPI. Similar to the findings on the co-primary efficacy endpoints, the pattern of response on all five secondary efficacy endpoints suggested non-significant improvements associated with bezisterim at most timepoints from baseline through the week 30 endpoint, with LS mean differences from placebo for each parameter as follows at week 30: ADCOMS, −0.03 (95% CI: −0.17, 0.12); ADL, 3.08 (95% CI: −1.28, 7.45); CGIC, −0.43 (95% CI: −1.22, 0.37); MMSE, 1.02 (95% CI: −1.01, 3.06); and NPI, −1.92 (95% CI: −5.65, 1.80) (Figure 2).

The completion data and results reported from 18-month clinical trials by approved medications van Dyck et al., (2023); Budd Haeberlein et al., (2022) are listed for comparison in Figure 2B.

### Safety and tolerability (per-protocol population)

Treatment-emergent adverse events (TEAEs) in the per-protocol population occurred in 72.7% (*n* = 24) of participants in the placebo group and 62.5% (*n* = 15) of participants in the bezisterim group. The TEAEs reported by ≥2 participants in either treatment group are listed in Table 3. The only TEAE occurring in ≥5% participants receiving bezisterim and higher than placebo was headache (bezisterim 12.5%; *n* = 3 vs. placebo 0%; *n* = 0). Treatment-related TEAEs occurred in 12.5% (*n* = 3) of participants in the bezisterim group and 18.2% (*n* = 6) of participants in the placebo group. Serious adverse events (SAEs) occurred in 4 participants (bezisterim, n = 1; *placebo, n* = 3); none were considered related to treatment. One SAE of pneumonia occurred in a bezisterim-treated participant. Seven SAEs occurred in the 3 placebo-treated participants: COVID-19, sepsis, tachycardia, pyrexia, bile duct stone, fall, and pelvic fracture. Three participants in the placebo group and none in the bezisterim group discontinued due to an adverse effect (AE).

**Table 3:** Adverse events occurring in at least two participants in either treatment group in the per-protocol population.

**TEAE, *n* (%)**	**Placebo (*n* = 33)**	**Bezisterim (*n* = 24)**
One or more TEAE	24 (72.7)	15 (62.5)
COVID-19	6 (18.2)	3 (12.5)
Headache	0	3 (12.5)
Fall	3 (9.1)	2 (8.3)
Urinary tract infection	3 (9.1)	2 (8.3)
Dizziness	2 (6.1)	1 (4.2)
Blood testosterone decreased	2 (6.1)	0
Gastroenteritis viral	2 (6.1)	0
Rash	2 (6.1)	0
Hypertension	2 (6.1)	0
Vomiting	2 (6.1)	0

TEAE, treatment emergent adverse event.

### Additional secondary endpoints and exploratory endpoints (per-protocol population)

**Figure 3:**
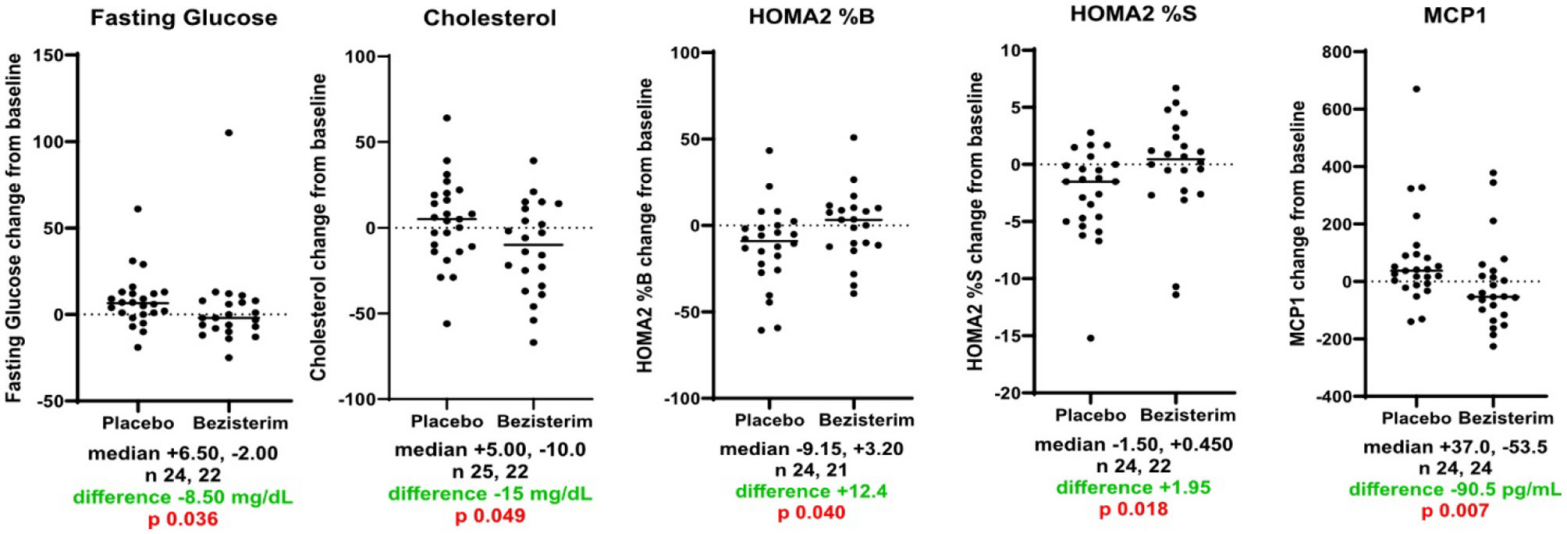
Additional secondary and exploratory endpoints (per-protocol population). Data distributions and median changes from baseline to completion visit are shown for the per-protocol participants. For additional secondary endpoints and exploratory endpoints, 2-sided exact Mann–Whitney nonparametric analyses were performed. For bezisterim HOMA2-%B, one extreme data point (330%) was excluded as an outlier (Grubbs test, at α = 0.05, G = 4.31; inclusion of this value further decreased the *p*-value to 0.024). The *p*-values were not corrected for multiple comparisons in this exploratory analysis. See abbreviations in the footnotes of Table 2.

The trial included additional objectives based on change from baseline values for metabolic, inflammatory, and dementia biomarkers. Figure 3 shows the data distributions and median changes from baseline to completion visit for the per-protocol participants. There were significant decreases in fasting glucose, cholesterol, and monocyte chemoattractant protein 1 (MCP1), and increases in HOMA2-%beta cell function and HOMA2-% insulin sensitivity associated with bezisterim compared to placebo. There were non-significant directional improvements in fasting insulin, HOMA2-IR, triglycerides, fructosamine, adiponectin, leptin, systolic and diastolic blood pressure, and weight (data not shown) associated with bezisterim. There were no significant differences in pTau, GFAP, NfL, or Aβ42/40 ratio (data not shown).

### Bezisterim treatment and epigenetic biological age (evaluable per-protocol population with completion of DNA methylation samples)

**Figure 4:**
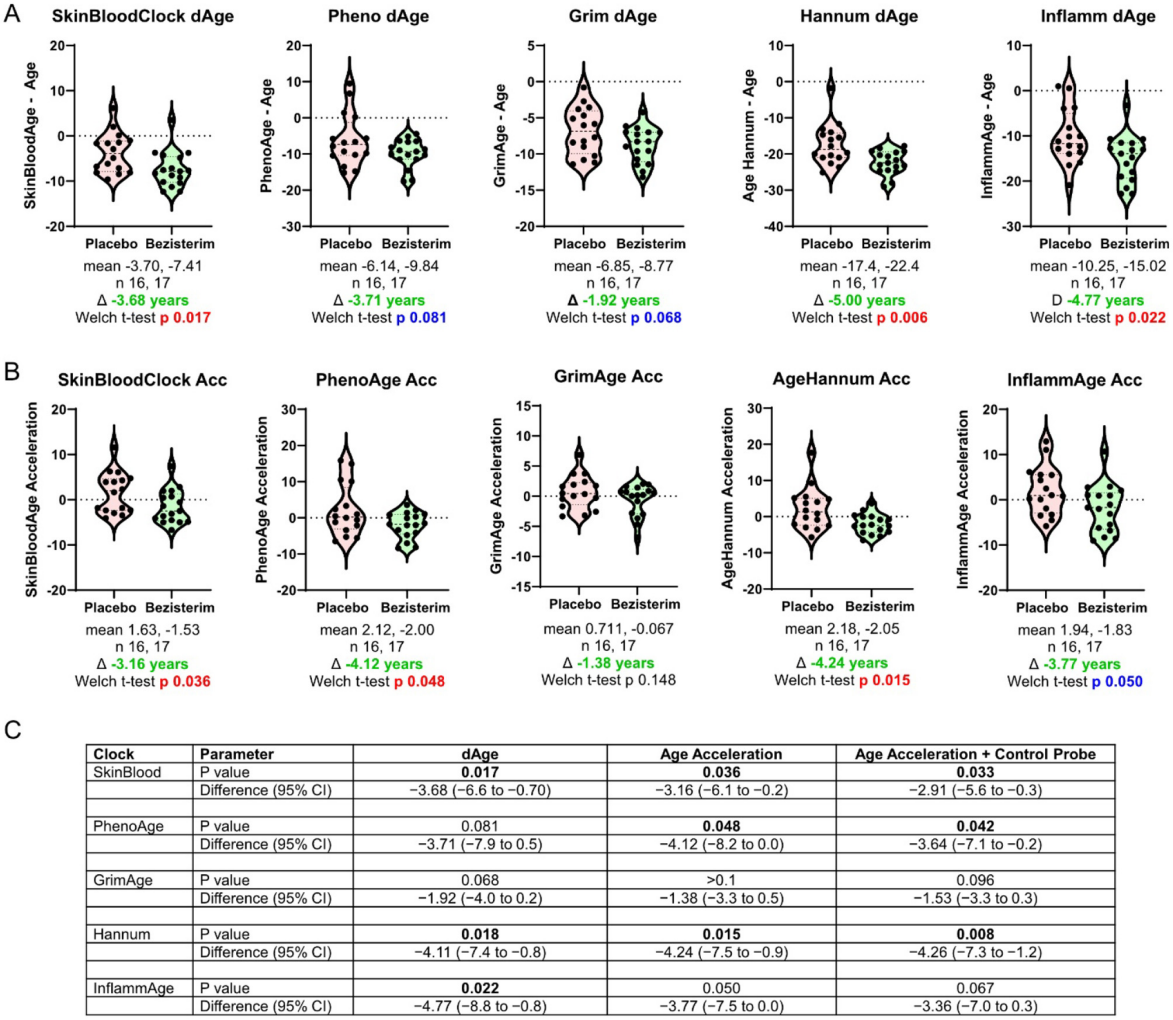
Difference between biologic clock age and chronologic age (per-protocol population with evaluable completion DNA methylation data). **(A)** Difference between biologic and chronological age (dAge) as calculated by different epigenetic aging clocks. **(B)** Age acceleration as calculated by different epigenetic aging clocks. **(C)** Clock corrections for dAge, age acceleration, and age and batch effects using control probes, Placebo (*n* = 16), Bezisterim (*n* = 17).

DNA methylation data were generated for 33 completion samples in the bezisterim (*n* = 17) and placebo (*n* = 16) groups. A suite of epigenetic clocks (SBCAge, PhenoAge, GrimAge, AgeHannum clock, and InflammAge) was used, and a value for dAge was derived. Overall, patients treated with bezisterim for 30 weeks displayed a higher improvement in biological age (lower dAge) compared to the placebo group across different epigenetic clocks (Figure 4). The average difference between the placebo and bezisterim groups was −3.68 years for SBCAge (*p* = 0.017), −3.71 years for PhenoAge (*p* = 0.081), −1.92 years for GrimAge (*p* = 0.068), −5.00 years for Hannum clock (*p* = 0.006), and −4.77 years for InflammAge (*p* = 0.022). While the improvement in dAge was only statistically significant for the SBC, Hannum clock, and InflammAge clock, all 5 clocks displayed a trend toward a reduction in dAge in bezisterim-treated patients. A consistent trend was observed when using age acceleration (similar to dAge but not confounded by chronological age, see “Methods” section) and when correcting for potential batch effects in the data using array control probes.

**Figure 5:**
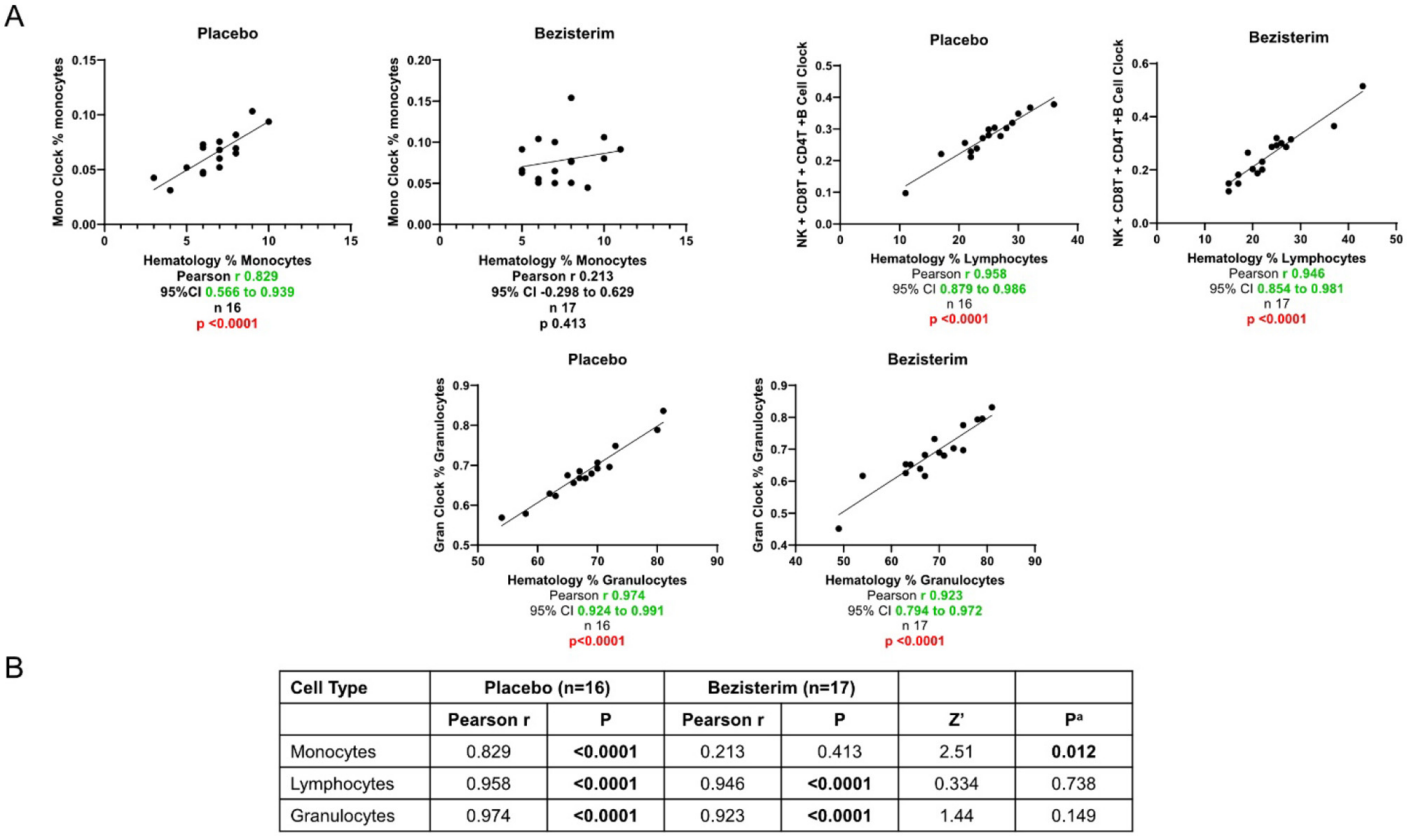
Change from baseline for blood cells (per-protocol population with evaluable completion DNA methylation data). **(A)** Graphs of the correlations. **(B)** Statistical analysis results of the correlations. ^a^Significance between correlations = Z test statistic of Fisher's to Z transformation.

There is a possibility that changes in cell composition may contribute to the observed differences in biological age between the 2 groups. Using Horvath's algorithms, we calculated the correlations between blood cell type populations (monocytes, lymphocytes, and granulocytes) measured with a clinical hematology analyzer and those calculated from DNA methylation data. Interestingly, with the exception of monocytes, all cell populations showed strong correlations after bezisterim treatment (Figure 5).

**Figure 6:**
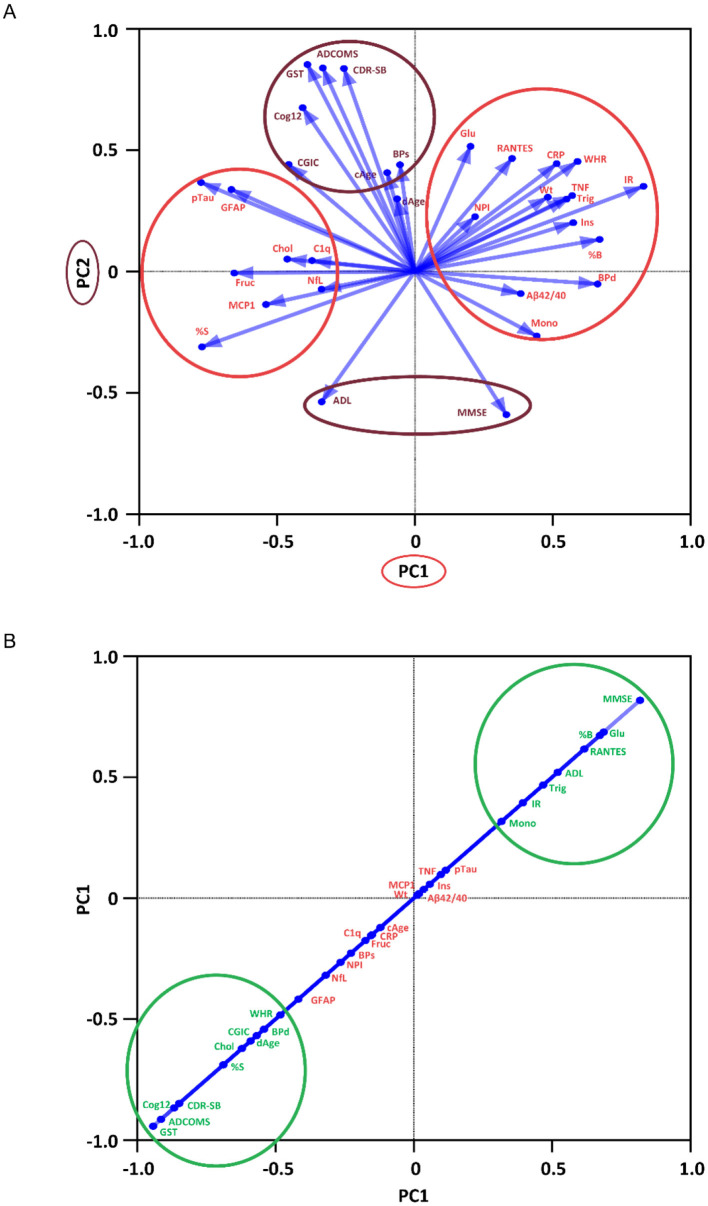
Principal component analysis of SkinBlood epigenetic aging clock results (per-protocol population with evaluable completion DNA methylation data). **(A)** Two principal components were identified for placebo (*n* = 16) correlations. **(B)** A single principal component was identified for bezisterim (*n* = 17) correlations.

The correlations of the SBC results and clinical measures were further examined using principal component analysis to reduce the data dimensionality (Figure 6). This analysis identified 2 principal components for correlations in placebo participants. The eigen vector clusters for metabolic, inflammatory, and dementia markers were distinct from the clusters for neurological assessments, and all eigen vectors except for fructosamine shared PC1 and PC2 contributions. The placebo dAge vector contributed least to the correlations. In distinction, there was only a single principal component identified for bezisterim correlations, combining major contributions from neurologic, metabolic, monocyte, regulated on activation, normal T-cell expressed and secreted (RANTES), and dAge vectors with minor contributions from inflammatory and dementia biomarkers, suggesting a potential homeostatic effect. The variance explained by each PC was 25.1% for placebo PC1 and 17.5% for placebo PC2. The cumulative variance was 42.5% for PC1 and PC2.

Examination of the bezisterim PC1 loadings >|0.5| indicated that increases in fasting glucose, pancreatic beta cell function, and the chemokine RANTES were associated with increases (improvements) in MMSE and ADL; and that decreases in SBC dAge, diastolic BP, cholesterol, and insulin sensitivity were associated with improvements (decreases) in CGIC, ADAS-Cog12, CDR-SB, ADCOMS, and GST. The placebo PC1 loadings >|0.5| indicated that increased waist-to-hip ratio (WHR), insulin resistance, diastolic BP, TNF, C-reactive protein (CRP), triglycerides, and insulin were correlated; and that increased MCP1, GFAP, fructosamine, insulin sensitivity, and pTau217 were associated with increase (decline) in CGIC. Placebo PC2 loadings above |0.5| indicated that increased CRP was associated with increases (declines) in CDR-SB, GST, ADCOMS, and Cog12; and that decreases (declines) in MMSE and ADL were correlated.

### Bezisterim treatment modified exploratory correlations between neurological assessments and metabolic, inflammatory, and AD biomarkers expected to be associated with improvement in AD

Seven AD biomarkers correlated with neurological assessments at study completion (Table 4). The table displays marked differences between the correlations for placebo subjects and bezisterim subjects. For placebo subjects, increases in GFAP were associated with a decline in cognitive and functional assessments (increased GST, CDR-SB, ADCOMS, ADAS-Cog 12; decreased MMSE). Increases in pTau217 were associated with decline (GST, ADCOMS, ADAS-Cog12, CGIC). Increased CRP levels were associated with a decline in ADL. RANTES levels were associated with a decline in CDR SB. There were no associations with SBC dAge for placebo subjects.

**Table 4:** Correlations between exploratory biomarkers and neurological assessments (per-protocol population).

	**GFAP**		**pTau**		**CRP**		**Chol**		**NfL**		**RANTES**		**SBC dAge**	
	**placebo**	**bezisterim**	**placebo**	**bezisterim**	**placebo**	**bezisterim**	**placebo**	**bezisterim**	**placebo**	**bezisterim**	**placebo**	**bezisterim**	**placebo**	**bezisterim**
GST	**0.67**	0.25	**0.44**	0.17	−0.04	0.02	−0.06	**0.5**	0.17	**0.48**	0.17	**−0.57**	0.21	0.47
CDRSB	**0.51**	0.27	**0.37**	0.13	0.37	−0.004	−0.02	0.39	0.15	**0.44**	**0.4**	**−0.45**	0.11	0.41
ADCOMS	**0.5**	0.29	**0.39**	0.17	0.002	−0.004	0.006	0.39	0.12	**0.45**	0.23	**−0.58**	0.13	0.47
Cog12	**0.59**	0.19	**0.41**	0.2	−0.17	0.041	−0.08	**0.55**	0.08	**0.46**	−0.03	**−0.64**	0.17	0.46
CGIC	0.3	0.23	**0.41**	0.21	−0.02	−0.08	0.32	0.18	0.32	0.14	−0.06	−0.39	0.02	0.47
ADL	−0.26	−0.28	−0.08	**−0.4**	**−0.46**	0.32	0.17	−0.37	−0.04	**−0.64**	−0.19	0.27	0.3	−0.08
MMSE	**−0.43**	−0.20	−0.29	−0.12	0.008	−0.22	0.02	**−0.47**	−0.19	−0.4	0.003	**0.52**	−0.01	**−0.58**

Associations were explored between eight biomarker measures and seven neurological assessments for placebo and bezisterim subjects. Pearson correlations with p < 0.05 (not corrected for multiple testing) are provided in bold in the table. Correlations of interest are highlighted in orange (positive) or blue (negative), see the text for details. Correlation analyses are from single laboratory or clinical measurements at the completion visit. Laboratory values are from single samples obtained from single blood draws at completion, but from different tubes depending on the test performed. pTau217, NfL, and GFAP data were from duplicate samples from the same tube for each subject. For placebo, n = 26 except for SBC dAge correlations (n = 16); for bezisterim, n = 24 except for SBC dAge correlations (n = 17).

For bezisterim subjects, decreased pTau was associated with improved ADL. Cholesterol (which was decreased in bezisterim subjects) was correlated with the decreases in GST and Cog12 and the increased MMSE scores. Improvements in GST, CDR SB, ADCOMS, Cog12, and ADL in bezisterim subjects were correlated with decreased NfL levels. For bezisterim subjects, RANTES was positively associated with improvements in GST, CDR SB, ADCOMS, Cog12, and MMSE scores. The reductions in SBC dAge (improvements) for bezisterim subjects were associated with numerical improvements in GST, CDR-SB, ADCOMS, ADAS-Cog12, CGIC, and MMSE (only the MMSE correlation with the decreased numbers of subjects with SBC dAge data was significant).

There was an evolution of the exploratory correlations in the associations between RANTES and CDR-SB over the 30-week treatment period for bezisterim participants (Table 5). At baseline, neither placebo nor bezisterim participants had significant correlations for these associations. Importantly, the difference between bezisterim baseline and completion was significantly different (*p* = 0.015). No significant change was observed in the placebo correlation over 30 weeks, and the difference in the correlation between placebo and bezisterim at completion was significant (*p* = 0.040).

**Table 5:** Exploratory Bezisterim-modified RANTES/CDR-SB correlations (per-protocol population).

**Treatment**	**Baseline**	**Completion**	**Fisher transformation**
Placebo	R 0.162, *p* = 0.438	R 0.146, *p* = 0.476	*p* = 0.956
Bezisterim	R 0.269, *p* = 0.204	R −0.445, ***p*** **=** **0.029**	***p*** **=** **0.015**
Fisher transformation	*p* = 0.710	***p*** **=** **0.040**	

^1^Baseline and completion *p* values for Pearson correlations between baseline and completion for placebo and for bezisterim.

^2^Fisher Z' transformation *p* values for Pearson correlations between baseline and completion.

^3^Fisher Z' *p* values are for transformed Pearson correlations between placebo and bezisterim at baseline and at completion.

Bold numbers indicate *p* < 0.05.

### Bezisterim treatment modified neurological assessment and its exploratory correlations with AD-related gene epigenetics

Neurological assessments and metabolic, inflammatory, and AD plasma biomarkers at study completion were tested for correlations at individual CpG sites, and the results were corrected for false discovery rate (FDR < 0.05) (Supplementary File S2). There were 24 CpG methylation sites with significant correlations to clinical measures. Significant correlations between clinical measures and CpG sites assigned to 18 different genes were observed in bezisterim-treated participants but not in placebo-treated participants. Significant correlations were observed between clinical measures and CpG sites assigned to six distinct genes (by genomic location) in placebo-treated participants but not in bezisterim-treated participants. The relationships of these genes to AD and references are detailed in Supplementary File S2 and are summarized in the following subsections.

#### Bezisterim-treated participant correlations

Methylation levels of CpG sites of 7 genes of interest in AD were correlated with WHR. G protein-coupled receptor 119 (GPR119) and vascular-binding protein 1 (VBP1) are involved in metabolic pathways regulating glucagon-like peptide-1 (GLP-1) and hypoxia-inducible factor 1-alpha (HIF-1α). Interleukin-1 receptor accessory protein-like 1 (IL1RAPL1) and TLR adaptor interacting with SLC15A4 (TASL) are involved in inflammatory signaling. Hephaestin (HEPH) is involved in the process of ferroptosis. RAB39B, a member of the RAS oncogene family, is important in autophagy and in the prevention of endoplasmic reticulum (ER) stress. Cleavage stimulation factor subunit 2 (CSTF2) plays a crucial role in neural and intellectual development.

Methylation of 2 CpG sites was associated with metabolic pathways involved in a PLAAT5/GPR119/GLP-1/HIF-1α/glucose metabolism pathway, where PLAAT5 refers to phospholipase A and acyltransferase 5. PLAAT5 cg23728669 methylation correlated with ADL scores. Both PLATT5 and GPR119 are involved in the metabolic regulator GLP-1 pathway. PLAAT5 synthesizes lipid amides, which are agonists of GPR119 that regulate GLP-1 synthesis.

Methylation of 4 CpG sites was related to HIF-1α. In addition to PLAAT5 and GPR119, ubiquitin-specific peptidase 22 (USP22), cg22310812, whose methylation correlates with NPI scores, plays a crucial role in regulating the NLR family pyrin domain containing 3 (NLRP3) inflammasome and HIF-1α regulation. Moreover, transmembrane protein 237 (TMEM237) is transactivated by HIF-1α. The TMEM237 promoter cg23849778 methylation correlated with ADCOMS scores, and TMEM237 variants are associated with ciliopathy and neurodevelopmental delays. In addition to the significant correlation with ADCOMS, the same TMEM237 CpG showed correlation trends with CDR-SB and with GST.

Methylation of 7 CpG sites was related to metabolic dysregulation. In addition to PLATT5 and GPR119, kelch-like family member 31 (KLHL31) cg25982641 methylation correlated with cholesterol levels. KLHL31 is involved in ubiquitination and inflammatory signaling. TGM6 cg02898994 methylation correlated with fasting glucose levels. Transglutaminase 6 (TGM6) is associated with glucose-stimulated insulin secretion, and a change in the methylation of this specific CpG site is associated with aging and an increased mortality risk. CDK5 and Abl enzyme substrate 1 (CABLES1) enhancer cg12892087 methylation correlated with MMSE scores. CABLES1 is a marker of glial cells and is activated in AD and PD in response to oxidative stress, aggregated proteins, and metabolic dysregulation. Zinc finger protein 33B (ZNF33B) cg21889224 methylation correlated with the ratio of monocytes to overall WBC count. ZNF33B is a transcriptional regulator associated with diabetes. Proline-rich coiled-coil 2A (PRRC2A) cg05182583 methylation correlated with MCP1 plasma levels. In addition to being related to the inflammatory processes of obesity and diabetes, PRRC2A is involved in neuromyelitis optica spectrum disorder.

Two CpG sites are associated with the deposition of neurotoxic protein. Discoidin domain receptor tyrosine kinase 1 (DDR1) cg01598675 methylation correlated with ADAS-Cog12 scores. DDR1 is important for triggering the expression of receptor expressed on myeloid cells 2 (TREM2), and for the reduction of neurotoxic proteins and inflammation. The target of EGR1, member 1 (TOE1) cg21853889 methylation was correlated with the homeostasis model assessment 2 of insulin sensitivity (HOMA2-%S). TOE1 regulates telomerase activity, and a decrease in this CpG in AD is also associated with increased tau deposition.

#### Placebo-treated participant correlations

Methylation of several CpG sites was associated with genes of interest in AD. Methylation of the catalase (CAT) promoter cg22159421 correlated with CRP. CAT is an antioxidant enzyme important in AD, PD, and other diseases of aging. In addition to the significant correlation with CRP, additional promoter CpG sites, cg06908474 and cg02564456, showed correlation trends with CRP. Methylation of non-specific cytotoxic cell receptor protein 1 (NCCRP1) cg21711545 correlated with CRP. NCCRP1 is involved in degradation of misfolded proteins.

Methylation of 2 CpG sites was associated with AD plasma biomarkers. Rubicon-like autophagy regulator (RUBCNL) enhancer cg02850812 methylation correlated with systolic blood pressure (BP). RUBCNL, like NCCRP1, is involved in the autophagy of protein aggregates in AD. IVD cg20278042 methylation correlated with the Aβ42/40 ratio. IVD is associated with AD risk, obesity, diabetes, loss of muscle mass, and frailty.

Methylation of 2 CpG sites was associated with immune and inflammatory signaling. Activating signal cointegrator 1 complex subunit 3 (ASCC3) promoter cg12253828 methylation was correlated with GFAP plasma levels. ASCC3 is involved in the regulation of signal transducer and activator of transcription 3 (STAT3) and interferon response genes, and variants are associated with developmental delays and increased pTau. Zinc finger protein 579 (ZNF579) enhancer cg14402955 methylation correlated with homeostasis model assessment 2 of β-cell function (HOMA2-%B). ZNF579 regulates the synthesis of inflammatory leukotriene, which is associated with AD neuroinflammation and cell death.

## Discussion

In the current study, bezisterim was associated with exploratory directional improvements in cognitive function in per-protocol participants with mild-to-moderate probable AD. The effect sizes for the per-protocol population primary and secondary neurological endpoints, which did not reach statistical significance, were comparable to those obtained with approved AD medications and were consistent in direction of improvement.

The exploratory analyses of primary and secondary neurological endpoints and biomarkers suggest that bezisterim treatment may improve neurological measures in AD, correlating with changes in clinical parameters and epigenetic and biological markers related to metabolism, oxidative stress, inflammation, aging, and dementia. Bezisterim treatment appeared to alter exploratory correlations compared to those found in placebo participants. While the directions of correlations observed in placebo-group participants were consistent with expectations for AD, directions of many of the correlations observed in bezisterim participants were consistent with the hypothesis that bezisterim-mediated improvements in inflammation driven by metabolic dysregulation might be associated with neurological improvements. For example, in placebo subjects, progression associated biomarkers, including GFAP, pTau217, and CRP levels at completion, were associated with cognitive and functional decline (as measured by GST, CDR-SB, ADCOMS, ADAS-Cog12, CGIC, ADL, and MMSE) as expected. For bezisterim subjects, however, the improvements relative to placebo in cognitive functional markers were associated with decreased cholesterol, NfL, SBC dAge, and increased RANTES.

RANTES is a chemokine reported to have contradictory roles, both neurodegenerative and neuroprotective, in AD (Li and Zhu, 2019; Ma et al., 2023) disease progression. While neither placebo nor bezisterim participants had significant correlations for these associations at baseline, at 7 months, placebo RANTES correlated with CDR SB decline, while the bezisterim participants' RANTES levels correlated with improvements in the GST, CDR-SB, ADCOMS, Cog12, and MMSE endpoints. These results are compatible with the hypothesis that RANTES might contribute to neurodegeneration over time, and that bezisterim treatment might alter regulatory effects of RANTES on cognitive assessments.

RANTES expression is dysregulated in AD and positively correlates with AD pathology, consistent with the placebo exploratory correlation with CDR-SB decline seen in this study (Li et al., 2023). It is possible that bezisterim treatment altered the RANTES interactions with microglial cells. RANTES also play neuroprotective roles (Tripathy et al., 2010), which is essential for hippocampal complex formation, learning, and memory (Ajoy et al., 2021), and is decreased with age in AD (Vacinova et al., 2021). Deficiency of the RANTES receptor CCR5 is associated with astrocyte activation, Aβ deposition, and impaired memory function (Lee et al., 2009; Hwang et al., 2016), and the nonfunctional human CCR5-delta32 polymorphism is associated with earlier dementia onset (Wojta et al., 2020). Discordant effects of RANTES in AD may possibly be explained by the RANTES binding and activating GPR75, a new target for metabolic syndrome and obesity (Murtaza et al., 2022), that may protect neuronal cells from Aβ toxicity (Ignatov et al., 2006) and regulate insulin secretion by pancreatic islet cells through activation of phosphoinositide 3-kinase (PI3K), protein kinase B (Akt), and MAP kinases (Liu et al., 2013).

Many AD risk genes are highly and/or selectively expressed by brain microglia and encode proteins critical to the clearance of Aβ by microglial phagocytosis. Accelerated aging (Thomas et al., 2022); proinflammatory NF-κB signaling (Pan et al., 2011); and metabolic dysfunction/deregulation of oxidative phosphorylation; anaerobic glycolysis (Piers et al., 2020); and glial insulin resistance (Alassaf and Rajan, 2023) disrupt Aβ phagocytosis, contributing to AD disease progression. The switching of microglia from a proinflammatory M1 state to an anti-inflammatory, restorative M2 (Laudati et al., 2017) state to induce microglial phagocytosis and clearance of Aβ (Ma et al., 2023; Lee et al., 2012) is highly dependent on microglial metabolism and disrupted by the inability to switch between oxidative phosphorylation and glycolysis. Considering the observed change in the monocyte clock vs the monocyte hematology results for bezisterim at study completion, which were similar to the results for changes from baseline to completion previously reported for the phase 2a results (Reading et al., 2023), it is possible that bezisterim facilitates this switch to the M2 phenotype in excessive proinflammatory environments driven by aging, metabolic dysregulation, and AD disease progression. Thus, in the correct context, when homeostasis is restored by affecting aging or metabolically driven inflammation, RANTES/CCR5, and potentially other biological markers with contradictory roles in AD, can have neuroprotective effects. Principal component analysis supports this notion. While 2 principal components were evident for placebo participants, all 31 clinical measures were included in a single principal component for bezisterim participants.

The analysis of bezisterim vs placebo treatment for the SBC results was in concert with the open-label phase 2a results of completion vs. baseline for bezisterim that were reported previously (Reading et al., 2023). The significant effect of bezisterim vs. placebo on the participant's dAge, as determined by multiple epigenetic clocks, and significant trends in correlations between improvement in age and neurological assessments in those treated with bezisterim but not placebo, suggest that biological age may be a modifiable factor in AD progression.

Analysis of the exploratory correlations between the clinical measures and CpG methylation sites yielded remarkable findings: Despite both groups showing exploratory correlations with different CpG methylation sites, all 18 bezisterim and all 6 placebo correlations were associated with pathways that may relate to neurodegeneration. Without expression studies, clarification of these differential exploratory correlations is lacking, but the identified CpG genes have been associated with AD (PRRC2A, IL1RAPL1, RUBCN, and IVD), aging (TGM6), metabolic dysregulation (PLAAT5, GPR119, and PRRC2A), oxidative stress (HEPH and CAT), and inflammation (IL1RAPL1). Interestingly, CpG sites linked to genes whose products affect the stability of HIF-1α (USP22, VBP1, and KCTD1) were among those identified. HIF-1α, which is the key controller of the metabolic response to hypoxia, has recently been implicated in the metabolic dysregulation and phagocytic dysfunction seen in microglia in amyloid plaques (March-Diaz et al., 2021) and the promotion of neuroinflammation in Parkinson's disease (Dong et al., 2024).

A reduction in the DNA methylation-based proxy for monocytes upon treatment (between baseline and completion) was also observed in a previous phase 2a bezisterim study (Reading et al., 2023). This data could indicate that bezisterim treatment is associated with an alteration of the monocyte methylome. There are similarities between the changes observed with bezisterim treatment and the recently discovered ETS proto-oncogene 2 transcription factor (ETS2), which is responsible for the macrophage inflammation program associated with many diseases of aging (Stankey et al., 2024), possibly through modulation of ERK2 docking effects on ETS transcription factors (Seidel and Graves, 2002; Piserchio et al., 2017).

Given that bezisterim binds ERK, and inhibits TLR4-driven ERK signaling, NF-κB activation, and TNF production, bezisterim may decrease inflammation-driven epigenetic changes that dysregulate inflammatory signaling. Indeed, NF-κB and TNF activation are known to increase DNA (cytosine-5)-methyltransferase 1 (DNMT1) and 3a, which are responsible for enhanced DNA methylation and histone regulation in aging and disease (Jiang et al., 2022; You et al., 2017; Pacaud et al., 2014; Guarnieri et al., 2020; Liu et al., 2012).

The findings in this study are severely limited by the small sample size with verified clinical and biomarker data. In addition, samples for DNA methylation analyses were not collected prior to treatment, thereby not allowing paired analyses for these findings. Data for the exploratory biomarker population are limited and will need replication in additional studies to validate and apply the trial findings. These limitations should not decrease the potential importance of these findings.

An anti-inflammatory insulin sensitizer that improves diseases of aging would be an important step forward in the battle against neurodegeneration. The data presented demonstrated consistent exploratory findings across varied analyses, suggesting a treatment effect. Unlike currently approved anti-amyloid drugs that have serious side effects, new therapies with the potential promise of anti-inflammatory and metabolic benefits and with reduced risks may decrease neurodegeneration. We need to pursue these leads in the challenging journey to decrease dementia.

The exploratory results suggest that bezisterim treatment altered the relationships between clinical findings and biological aging, and metabolic, inflammatory, dementia, and individual epigenetic biomarkers. Correlations between clinical measures and DNA methylation of specific CpG sites of AD-related genes may also suggest that the anti-inflammatory activity of bezisterim is involved in the regulation of gene expression related to neurodegeneration, biological aging, oxidative stress, dementia, metabolic, and inflammatory biomarkers in AD progression. Future studies of bezisterim in dementia in well-controlled clinical studies will be required to confirm these exploratory findings. Bezisterim might prove useful for other diseases of aging beyond AD.

## Data Availability

The datasets presented in this study can be found in online repositories. The names of the repository/repositories and accession number(s) can be found below: https://doi.org/10.5281/zenodo.13984028, 13984028.
