# Correction: An exploratory analysis of bezisterim treatment associated with decreased biological age acceleration, and improved clinical measure and biomarker changes in mild-to-moderate probable Alzheimer's disease

**DOI:** 10.3389/fnins.2025.1758523

**Published:** 2026-01-12

**Authors:** Christopher L. Reading, Jiayan Yan, Marcia A. Testa, Donald C. Simonson, Hira Javaid, Lisa Schmunk, Daniel E. Martin-Herranz, Robert Brooke, Juozas Gordevicius, Jeffrey Zhang, Harvey Yuan, Clarence Ahlem, Lixia Wang, Penelope Markham, Nily Osman, Stephen O'Quinn, Joseph Palumbo

**Affiliations:** 1 BioVie Inc., Carson City, NV, United States; 2 Department of Biostatistics, Harvard T. H. Chan School of Public Health, Boston, MA, United States; 3 Division of Endocrinology, Diabetes, and Hypertension, Brigham and Women's Hospital, Harvard Medical School, Boston, MA, United States; 4 Hurdle.bio/Chronomics Ltd., London, United Kingdom; 5 Epigenetic Clock Development Foundation, Torrance, CA, United States; 6 Princeton Pharmatech, Princeton, NJ, United States

**Keywords:** Alzheimer's, neuroinflammation, insulin, aging, DNA methylation, bezisterim, cognition, dementia

There was a mistake in Figure 7 and the corresponding caption as published. The authors discovered and corrected errors in Figure 7, as described in further detail below. After removing the pseudocolor heat maps from the figure, the remaining display items were renamed as Table 4 and the corresponding text in the caption was revised to capture the extent of revisions. The corrected caption of Figure 7, renamed as Table 4, appears below. The prior Table 4: Exploratory Bezisterim-modified RANTES/CDRSB correlations (per-protocol population) remains unedited.

Table 4; Correlations between exploratory biomarkers and neurological assessments (per-protocol population) caption now reads as follows. Associations were explored between eight biomarker measures and seven neurological assessments for placebo and bezisterim subjects. Pearson correlations with *p* < 0.05 (not corrected for multiple testing) are provided in bold in the table. Correlations of interest are highlighted in orange (positive) or blue (negative), see the text for details. Correlation analyses are from single laboratory or clinical measurements at the completion visit. Laboratory values are from single samples obtained from single blood draws at completion, but from different tubes depending on the test performed. pTau217, NfL, and GFAP data were from duplicate samples from the same tube for each subject. For placebo, *n* = 26 except for SBC dAge correlations (*n* = 16); for bezisterim, *n* = 24 except for SBC dAge correlations (*n* = 17).

There was a mistake in Figure 7 as published. Errors were detected in the signs, correlations, and *P* values used during the heat map formation. We removed the heat maps to eliminate the visualization of the errors, corrected the data in the remaining table, rearranged the table to facilitate side-by-side between-group comparisons, and renamed the display item to Table 4: Correlations between exploratory biomarkers and neurological assessments in the per-protocol population. The corrected table, appears below.

**Table 4:** Correlations between exploratory biomarkers and neurological assessments (per-protocol population).

	**GFAP**		**pTau**		**CRP**		**Chol**		**NfL**		**RANTES**		**SBC dAge**	
	**placebo**	**bezisterim**	**placebo**	**bezisterim**	**placebo**	**bezisterim**	**placebo**	**bezisterim**	**placebo**	**bezisterim**	**placebo**	**bezisterim**	**placebo**	**bezisterim**
GST	**0.67**	0.25	**0.44**	0.17	−0.04	0.02	−0.06	**0.5**	0.17	**0.48**	0.17	**−0.57**	0.21	0.47
CDRSB	**0.51**	0.27	**0.37**	0.13	0.37	−0.004	−0.02	0.39	0.15	**0.44**	**0.4**	**−0.45**	0.11	0.41
ADCOMS	**0.5**	0.29	**0.39**	0.17	0.002	−0.004	0.006	0.39	0.12	**0.45**	0.23	**−0.58**	0.13	0.47
Cog12	**0.59**	0.19	**0.41**	0.2	−0.17	0.041	−0.08	**0.55**	0.08	**0.46**	−0.03	**−0.64**	0.17	0.46
CGIC	0.3	0.23	**0.41**	0.21	−0.02	−0.08	0.32	0.18	0.32	0.14	−0.06	−0.39	0.02	0.47
ADL	−0.26	−0.28	−0.08	**−0.4**	**−0.46**	0.32	0.17	−0.37	−0.04	**−0.64**	−0.19	0.27	0.3	−0.08
MMSE	**−0.43**	−0.20	−0.29	−0.12	0.008	−0.22	0.02	**−0.47**	−0.19	−0.4	0.003	**0.52**	−0.01	**−0.58**

*Associations were explored between eight biomarker measures and seven neurological assessments for placebo and bezisterim subjects. Pearson correlations with p*<*0.05 (not corrected for multiple testing) are provided in bold in the table. Correlations of interest are highlighted in orange (positive) or blue (negative), see the text for details. Correlation analyses are from single laboratory or clinical measurements at the completion visit. Laboratory values are from single samples obtained from single blood draws at completion, but from different tubes depending on the test performed. pTau217, NfL, and GFAP data were from duplicate samples from the same tube for each subject. For placebo, n* = *26 except for SBC dAge correlations (n* = *16); for bezisterim, n* = *24 except for SBC dAge correlations (n* = *17)*.

In the original article, there was an error. The results summary for the data that were shown in Figure 7 paralleled the errors that were noted above for Figure 7.

A correction has been made to **Results**, Bezisterim treatment modified exploratory correlations between neurological assessments and metabolic, inflammatory, and AD biomarkers expected to be associated with improvement in AD, paragraph numbers 1 and 2:

Seven AD biomarkers correlated with neurological assessments at study completion (Table 4). The table displays marked differences between the correlations for placebo subjects and bezisterim subjects. For placebo subjects, increases in GFAP were associated with a decline in cognitive and functional assessments (increased GST, CDR-SB, ADCOMS, ADAS-Cog 12; decreased MMSE). Increases in pTau217 were associated with decline (GST, ADCOMS, ADAS-Cog12, CGIC). Increased CRP levels were associated with a decline in ADL. RANTES levels were associated with a decline in CDR SB. There were no associations with SBC dAge for placebo subjects.

For bezisterim subjects, decreased pTau was associated with improved ADL. Cholesterol (which was decreased in bezisterim subjects) was correlated with the decreases in GST and Cog12 and the increased MMSE scores. Improvements in GST, CDR SB, ADCOMS, Cog12, and ADL in bezisterim subjects were correlated with decreased NfL levels. For bezisterim subjects, RANTES was positively associated with improvements in GST, CDR SB, ADCOMS, Cog12, and MMSE scores. The reductions in SBC dAge (improvements) for bezisterim subjects were associated with numerical improvements in GST, CDR-SB, ADCOMS, ADAS-Cog12, CGIC, and MMSE (only the MMSE correlation with the decreased numbers of subjects with SBC dAge data was significant).

In the original article, there was an error. The discussion of the data that are summarized by Figure 7 included errors that paralleled those that were noted above for Figure 7.

A correction has been made to **Discussion**, Paragraph Numbers 2–3:

The exploratory analyses of primary and secondary neurological endpoints and biomarkers suggest that bezisterim treatment may improve neurological measures in AD, correlating with changes in clinical parameters and epigenetic and biological markers related to metabolism, oxidative stress, inflammation, aging, and dementia. Bezisterim treatment appeared to alter exploratory correlations compared to those found in placebo participants. While the directions of correlations observed in placebo-group participants were consistent with expectations for AD, directions of many of the correlations observed in bezisterim participants were consistent with the hypothesis that bezisterim-mediated improvements in inflammation driven by metabolic dysregulation might be associated with neurological improvements. For example, in placebo subjects, progression associated biomarkers, including GFAP, pTau217, and CRP levels at completion, were associated with cognitive and functional decline (as measured by GST, CDR-SB, ADCOMS, ADAS-Cog12, CGIC, ADL, and MMSE) as expected. For bezisterim subjects, however, the improvements relative to placebo in cognitive functional markers were associated with decreased cholesterol, NfL, SBC dAge, and increased RANTES.

RANTES is a chemokine reported to have contradictory roles, both neurodegenerative and neuroprotective, in AD (Li and Zhu, 2019; Ma et al., 2023) disease progression. While neither placebo nor bezisterim participants had significant correlations for these associations at baseline, at 7 months, placebo RANTES correlated with CDR SB decline, while the bezisterim participants' RANTES levels correlated with improvements in the GST, CDR-SB, ADCOMS, Cog12, and MMSE endpoints. These results are compatible with the hypothesis that RANTES might contribute to neurodegeneration over time, and that bezisterim treatment might alter regulatory effects of RANTES on cognitive assessments.

The original article has been updated.

