# Supplementary material for: An exploratory analysis of bezisterim treatment associated with decreased biological age acceleration, and improved clinical measure and biomarker changes in mild-to-moderate probable Alzheimer's disease
Source: Front Neurosci. 2025 May 2;19:1516746. doi: 10.3389/fnins.2025.1516746 (PMC12082838; doi:10.3389/fnins.2025.1516746)
Supplement: Supplementary file 1 [file Data_Sheet_1.docx]

Supplementary File 1

Additional CONSORT information for the overall trial

The clinical results of NM101 participants were disclosed at AD/PD™ 2024 March 5-9, in Lisbon, Portugal, and at the 12th Alzheimer’s & Parkinson’s Drug Development Summit, April 23-25, 2024, in Boston, MA. Additional CONSORT information for the overall trial is summarized below.

There were important protocol amendments with changes to safety, eligibility, or study endpoints. Version 1.1 (14 April 2021) excluded females of child-bearing potential as bezisterim did not have supporting reproductive toxicology data. Version 1.2 (20 April 2022) added exclusion of recent COVID-19 infection that could impact neurological testing. Version 1.2 also added inclusion criterion of historical MRI or CT scan to confirm absence of pathobiology that might account for the cognitive disorder and historical evidence of cognitive impairment or dementia diagnosis, and removal of inclusion requirement for evidence of Aβ, since bezisterim was not an anti-amyloid treatment, and enrollment was very challenging for the Aβ positive population. Version 1.3 (05 Dec 2022) revised continuous glucose monitoring to be optional, as it was perceived that it might be limiting enrollment. Version 1.4 (10 Mar 2023) added pTau, GFAP, NfL, adiponectin, leptin and DNA methylation analysis to biomarkers to better characterize the response correlations. Version 2.0 (12 May 2023) changed the primary endpoint to co-primary endpoints of ADAS-Cog12 and ADCS-CGIC to a single primary endpoint of CDR-SB, based on recent endpoints used in AD trials. Version 3.0 (09 October 2023) changed the primary efficacy endpoints to co-primaries of ADAS-Cog12 and ADCS-CGIC, based on discussions with the US Food and Drug Administration (FDA) that a single primary endpoint would not be adequate for accelerated approval for agents without an established biomarker. The protocol was also modified to include the statistical considerations in the SAP prior to unblinding, including the mITT and per-protocol populations definitions, and clarifications of the secondary, tertiary and exploratory endpoints. Version 3.1 (23 October 2023) was modified to change the co-primaries to CDR-SB and ADAS-Cog12 to be more in line with recent approvals based on CDR-SB.

In version 3.1, the sample size was changed to approximately 400 subjects to be randomized into the study with a 1:1 treatment ratio in order to have at least 80% to detect the treatment difference of co-primary endpoints. This was estimated from the CDR-SB change from baseline results from the mild AD patients of our open label study (MMSE >20, n= 18). Conservatively, we assume that the treatment group would observe −0.46 CDR-SB improvement (2/3 of −0.7 CDR-SB improvement observed in the open label study). Assuming a placebo effect contributed approximately −0.23 of the −0.46 CDR-SB point improvement and that placebo would decline (CDR-SB increase) 0.15 during the study, the net assumed treatment difference was 0.38. Assuming the same standard deviation observed in the open label study, SD = 1.1. As for ADAS-Cog12, the samples were estimated to have at least 80% power to detect a 2.1-point difference between bezisterim and placebo assuming a SD of 7. Placebo is expected to have a similar SD.

All changes to the study were reviewed by the Sponsor and CRO who were blinded to treatment allocations. Although an interim analysis was originally planned, the rapid enrollment following the protocol modifications made this impractical, and the interim was eliminated.
